# A Visit to Foreign Dental Schools and Other Observations

**Published:** 1886-08

**Authors:** A. W. Harlan

**Affiliations:** Chicago, Illinois


### Independent Practitioner.

Vol. VII. August, 1886. No. 8.

### Original Communications.

Note.—No paper published or to be published in another journal will be accepted for this department. All papers must be in the hands of the Editor before the first day of the month preceding that in which they are expected to appear. Extra copies will be furnished to each contributor of an accepted original article, and reprints, in pamphlet form, may be had at the cost of the paper, press-work and binding, if ordered when the manuscript is forwarded. The Editor and Publishers are not responsible for the opinions expressed by contributors. The journal is issued promptly, on the first day of each month.

### A VISIT TO FOREIGN DENTAL SCHOOLS AND OTHER OBSERVATIONS.

BY A. W. HARLAN, M. D., D. D. S., CHICAGO, ILLINOIS.

(Continued from page 235.)

The operating room is generally called a "surgery," and is usually situated at some distance from the waiting room, so that no unpleasant odors are detected, or sounds heard by the patient who is next to be operated upon. As most dentists are located in residences, this is generally easy of accomplishment, for our free American method of receiving patients has not been adopted in Europe. The caller is usually conducted to the waiting room by a servant in evening dress. In some establishments I noticed a visitor's register, where the name of the patient is either written by himself or the attendant. I consider this a good idea, as at home many callers do not give their name or present a card, and hence their identity is not disclosed until they enter the operating room. If some such system prevailed with us we could escape many an interruption from transient callers, whose only aim is to sell something, or to inquire about fees, etc.

The dental laboratory is called a "workshop," which it generally resembles, as it is frequently located in the basement, and is supplied, in many instances, with ponderous machinery. Some of these "workshops" are very large, as many artificial teeth are made in England. If the proprietor does a good business, you will find a foreman and several workmen and boys seated at benches, or polishing plates, or engaged in the act of putting up cases or "orders" of teeth. To undertake the manufacture of a set of teeth is called taking an "order." The dentist, or his assistant, takes the impression and "bite," and the work is done below. I believe that the "workman" never sees the patient, except in rare instances. Some of them receive good pay, and they work long hours, about ten or eleven per day. They usually enter the establishment from a side street or alley, and never at the front door. Very few workmen ever attain to the dignity of a registered dentist. Some of them practice dentistry under the name of a druggist or chemist, who got his name registered as a dentist by the act of 1878, because he extracted teeth when the English law went into effect. Many unqualified men in this manner figure as dentists, who really have no right to call themselves such.

In a large city, like London, there are many dental "workshops" not connected with practices, where artificial teeth are made for dentists in regular practice, and I understand that the proprietors of some of them practice the art of mechanical dentistry for the middle and lower classes without being found out by the authorities. From my own observations, there are a good many men practicing dentistry in England whose names are not on the register. Many of these are Americans. They have charge of branch offices, which are quite numerous in London and the suburbs. Most of the Americans thus practicing, either as assistants or in charge of branches, are graduates of American Colleges not recognized by the English Medical Council. In this way English dentists, who may not be expert operators, employ the skill of native "Yankees," and sometimes their cards will say "American operators in constant attendance," etc. The chief ambition of many English dentists is to get on the attending or consulting staff of a hospital or infirmary, because after this is accomplished their fortune is assured. (We Americans are not so slow in this direction.) Professional men everywhere are not likely to throw such opportunities away, when they generally lead to acquaintance with medical men and the public at large.

I was rather astonished to find so few society organizations in England. The British Dental Association, it is true, meets annually, and there are several branches in various parts of the kingdom, but in London there is practically but one society—the Odontological. A students' society is connected with each of the schools, and they have the Odonto-Chirurgical Society of Scotland, at Edinburgh, but I believe there is no society in Wales or Ireland, unless there be one in Dublin. The membership of the Odontological Society is not large—about one hundred and twenty I believe. There is no American Dental Society in London. There is room for one, and also for two or three native societies. The Association of Surgeons practicing Dentistry is defunct, or if not, their proceedings are no longer published. It is expected that several new branches of the British Dental Association will be established very soon. There ought to be a society in every large city, at least in Great Britain. Liverpool, Manchester, Leeds, Sheffield, Glasgow, Dublin, Belfast, and other cities not so large, could sustain societies, if eight or ten men would go to work and shake off little differences of opinion, which are many times only imaginary, and thus raise the standard by associated effort. Great Britain needs a few missionary dentists to wake up the indolent and routine practitioners. When this is done, more valuable ideas will come to light than many now dream of. It will be a good thing for the people, as more natural teeth will be saved, and after all that is what we hope to accomplish.

Dental journalism is represented by three publications. "The Journal of the British Dental Association" is edited by the accomplished Underwood, a genuinely scientific man, who has already made of his journal a very readable and welcome periodical. It is a monthly, and the subscription price is only about $1.80 per annum. "The Dental Record" is edited by Thos. Gaddes, one of the veteran teachers of the National Dental Hospital, and its Dean. It is also a monthly, and the subscription price is the same. I have been a subscriber to it from its first issue, and can recommend it to my American confrères. Last, but not least, is the old "British Journal of Dental Science," a semi-monthly, issued the first and fifteenth of every month. This is edited impersonally, though if so minded I could give the editor's name. I do not propose to say which I think is the best journal, for they have all treated me better than I deserve, by republication of my own articles at various times. The price of the last named is double that of either of the others, but it must be remembered that it appears twice to each of the other's once. I could not be satisfied if I did not see its blue cover on my library table every fortnight from year to year. I am a believer in dental periodicals, and very often in former years it has only been by close economy that I could take them all. I have never regretted the sacrifice, as I have usually found something of value even in the smallest and most insignificant of the quarterly advertising sheets. I recommend more of the American dentists at home to become subscribers to at least one of the journals named, as it will well repay them for the outlay, and they will thereby see how great a world lies beyond their own horizon.

In closing this paper on the customs and habits and other things seen in Great Britain, a word or two must be said about the dental depots. We are apt to think that they are very much behind us in producing novelties in the way of new instruments and appliances. This is a mistake. There are several large establishments in London whose sales of goods of their own manufacture must be enormous. Albion is the land of cements, amalgams, artificial teeth, nitrous oxide, rubbers, forceps, and modeling compounds. Many American inventions are brought out in England almost as soon as they are sold here. One reason why so many goods of American manufacture are sold in England, is because there is no tariff. Our prohibitory tariff laws, on the other hand, prevent the English merchants and manufacturers from introducing their goods into this country, by reason of the excessive duties levied upon them. When we are not certain that we are to get good value for our money, we do not invest in goods of foreign workmanship, and as the duties are so high, there is scarcely any profit to the dealer who might keep English goods; hence, we seldom see articles of English dental merchandise. I will say for the products of the English dental manufactories, that I have found them in many instances as good in quality, and in some cases much better than our own, and in spite of the heavy duties, quite as cheap as American products.

Every little device which is evolved from the brain of the dentist is not patented in Great Britain as often as it is at home, and in consequence many small articles do not have fancy prices attached to them. I cannot commend the practice of keeping secret the components of many medicaments which are offered for sale abroad as well as at home, as this sort of quackery is not in keeping with our desire to be considered a learned profession. The habit prevails in England and the United States unfortunately, by some would-be scientific gentlemen, of offering secret anæsthetics, obtunders and other panaceas, at so much per bottle, and when they are advertised in dental journals it gives them a certain value to the illiterate and unthinking, and thereby degrades the profession of which they are members. If the publishers could exclude such advertisements, empirical and quackish nostrums would soon fade out of existence. In the next number I will give my impression of Germany and German dentists.

